# Endogenous cortisol levels are associated with an imbalanced striatal sensitivity to monetary *versus* non-monetary cues in pathological gamblers

**DOI:** 10.3389/fnbeh.2014.00083

**Published:** 2014-03-25

**Authors:** Yansong Li, Guillaume Sescousse, Jean-Claude Dreher

**Affiliations:** ^1^Reward and Decision Making Team, Centre de Neurosciences Cognitives, CNRS, UMR 5229Lyon, France; ^2^Neuroscience Department, Université Claude Bernard Lyon 1Lyon, France

**Keywords:** cortisol, reward, pathological gambling, fMRI, ventral striatum, addiction, incentive, glucocorticoid hormones

## Abstract

Pathological gambling is a behavioral addiction characterized by a chronic failure to resist the urge to gamble. It shares many similarities with drug addiction. Glucocorticoid hormones including cortisol are thought to play a key role in the vulnerability to addictive behaviors, by acting on the mesolimbic reward pathway. Based on our previous report of an imbalanced sensitivity to monetary versus non-monetary incentives in the ventral striatum of pathological gamblers (PGs), we investigated whether this imbalance was mediated by individual differences in endogenous cortisol levels. We used functional magnetic resonance imaging (fMRI) and examined the relationship between cortisol levels and the neural responses to monetary versus non-monetary cues, while PGs and healthy controls were engaged in an incentive delay task manipulating both monetary and erotic rewards. We found a positive correlation between cortisol levels and ventral striatal responses to monetary versus erotic cues in PGs, but not in healthy controls. This indicates that the ventral striatum is a key region where cortisol modulates incentive motivation for gambling versus non-gambling related stimuli in PGs. Our results extend the proposed role of glucocorticoid hormones in drug addiction to behavioral addiction, and help understand the impact of cortisol on reward incentive processing in PGs.

## Introduction

Glucocorticoid hormones (cortisol in humans and corticosterone in rodents) are produced by the adrenal cortex after the hypothalamic-pituitary-adrenal (HPA) axis is stimulated by psychologically or physiologically arousing stimuli (Sapolsky et al., 2000; Herman et al., 2005; Ulrich-Lai and Herman, 2009). These hormones have essential roles in normal physiological processes, such as acting on anti-stress and anti-inflammatory pathways, and, by doing so, have wide-ranging effects on behavior. Over the past few years, the potential role of glucocorticoid hormones on mental disorders has gained increased attention (Meewisse et al., 2007; Wingenfeld and Wolf, 2011). In particular, in the search for risk factors for drug addiction, increasing evidence points to an interaction between HPA functioning and drug exposure (Stephens and Wand, 2012). For example, a positive correlation between glucocorticoid levels and self-administration of psychostimulants has been observed in rodents (Goeders and Guerin, 1996; Deroche et al., 1997). In addition, drug administration produces stress-like cortisol responses (Broadbear et al., 2004) and similarly, acute administration of cortisol promotes cocaine craving in cocaine-dependent individuals (Elman et al., 2003). These findings not only point to the link between glucocorticoid hormones and addiction (Lovallo, 2006), but also emphasize the need to develop integrative theories explaining the mechanisms by which they affect addictive behavior.

Animal and human neuroimaging studies have demonstrated that addiction involves altered functioning of the mesolimbic reward system (Koob and Le Moal, 2008; Koob and Volkow, 2010; Schultz, 2011). Another line of research has shown that altered HPA response is associated with changes in dopaminergic regulation (Oswald and Wand, 2004; Alexander et al., 2011) and that glucocorticoid hormones have modulatory effects on dopamine release in the mesolimbic pathway, especially in the nucleus accumbens (NAcc; Oswald et al., 2005; Wand et al., 2007). Building on this evidence, it has been proposed that glucocorticoid hormones have facilitatory effects on behavioral responses to drugs of abuse, and that these effects are implemented *via* action on the mesolimbic reward system (Marinelli and Piazza, 2002; de Jong and de Kloet, 2004). Furthermore, on the basis of the incentive sensitization theory stating that the mesolimbic reward system mediates addiction-related cue hypersensitivity (Robinson and Berridge, 1993; Vezina, 2004, 2007; Robinson and Berridge, 2008), it has been proposed that glucocorticoid hormones contribute to drug addiction by modulating this neural system directly (Goodman, 2008; Vinson and Brennan, 2013).

Pathological gambling is a behavioral addiction characterized by compulsive gambling behavior and loss of control, which has gained much attention recently (van Holst et al., 2010; Conversano et al., 2012; Achab et al., 2013; Clark and Limbrick-Oldfield, 2013; Petry et al., 2013; Potenza, 2013). Since pathological gambling behavior shares many similarities with drug addiction in terms of clinical phenomenology (e.g., craving, tolerance, compulsive use, or withdrawal symptoms), heritability, and neurobiological profile (Potenza, 2006, 2008; Petry, 2007; Wareham and Potenza, 2010; Leeman and Potenza, 2012), it may be similarly under the influence of glucocorticoid hormones. However, little is known about the interaction between glucocorticoid hormones and incentive reward processing in pathological gambling. In the present study, we examined how endogenous cortisol modulates the processing of monetary and non-monetary cues in PGs. To achieve this goal, we re-analyzed previously published data using an incentive delay task manipulating both monetary and erotic rewards in PGs and healthy controls (Sescousse et al., 2013), and performed further correlation analyses between basal cortisol levels and neural responses. Based on the role of glucocorticoid hormones in drug addiction, we expected endogenous cortisol levels to be associated with neural responses to addiction-related cues versus non-addiction related cues. Specifically, since our previously published analysis found a differential response to monetary versus erotic cues in the ventral striatum of gamblers (Sescousse et al., 2013), we expected that higher cortisol levels would be associated with an increased differential response in anticipation of monetary versus erotic rewards in PGs.

## Materials and methods

### Subjects

We evaluated 20 healthy control subjects and 20 PGs. All were right-handed heterosexual males. We chose to study only men because men generally respond more to visual sexual stimuli than women (Hamann et al., 2004; Rupp and Wallen, 2008) and because there is a higher prevalence of pathological gambling among men than among women (Blanco et al., 2006; Kessler et al., 2008). The dataset from these subjects has already been used in our published functional magnetic resonance imaging (fMRI) study aiming at comparing primary and secondary rewards in healthy controls and pathological gamblers (PGs; Sescousse et al., 2013). Our current analysis focuses specifically on the relationship with cortisol levels and is therefore entirely original. As described in Sescousse et al. (2013), our published analysis excluded data from two PGs, due to technical problems with the task presentation in one case, and due to a highly inconsistent behavior in terms of hedonic ratings throughout the task in the other case. In the current analysis, we further discarded the data from one pathological gambler, because of a failure in successfully collecting blood samples. Therefore, the results reported are based on 20 healthy control subjects and 17 PGs. All subjects gave written informed consent to participate in the experiment. The study was approved by the local ethics committee (Centre Léon Bérard, Lyon, France).

Subjects underwent a semi-structured interview (Nurnberger et al., 1994) performed by a psychiatrist. All PGs met the DSM-IV-TR [Diagnostic and Statistical Manual of Mental Disorders (fourth edition, text revision)] criteria for pathological gambling diagnosis. Patients had a minimum score of 5 on the South Oaks Gambling Screen questionnaire (SOGS; range: 5–14) (Lesieur and Blume, 1987). Importantly, all were active gamblers, and none were under therapy or treatment of any type. Healthy control subjects had a score of 0 on the SOGS questionnaire, except one subject who had a score of 1. In both groups, a history of major depressive disorder or substance abuse/dependence (except nicotine dependence) in the past year was considered an exclusion criterion. All other DSM-IV-TR axis I disorders were excluded based on lifetime diagnosis.

We used a number of questionnaires to assess our subjects. The Fagerstrom Test for Nicotine Dependence (FTND; Heatherton et al., 1991) measured their nicotine dependence severity; the Alcohol Use Disorders Identification Test (AUDIT; Saunders et al., 1993) was employed to estimate their alcohol consumption; the Hospital Anxiety and Depression scale (HAD; Zigmond and Snaith, 1983) was used to evaluate current depressive and anxiety symptoms; and finally the Sexual Arousability Inventory (SAI; Hoon and Chambless, 1998) was used to assess their sexual arousal. Both groups were matched on age, nicotine dependence, education, alcohol consumption, and depressive symptoms (Table 1). PGs scored slightly higher on the anxiety subscale of the HAD questionnaire. Importantly, the two groups did not differ on income level and sexual arousability (Table 1), thereby ensuring a comparable motivation across groups for monetary and erotic rewards.

**Table 1:** **Demographic and clinical characteristics of PGs and healthy controls**.

	**Healthy control subjects (*n* = 20)**	**Pathological gamblers (*n* = 17)**	**Group comparison**
Age	31±7.3	34±11.9	*U* = 157.5, *p* = 0.71
Education level (number of years)	13.2±1.7	12±2.7	*U* = 132, *p* = 0.24
Monthly income (€)	1537.5±1010.7	2191.2±1410.2	*U *= 124.5, *p* = 0.16
SAI	88.6±12.6	92.5±14.8	*t*_(35)_ = 0.89, *p* = 0.38
AUDIT	4.2±3.5	6±4	*t*_(35)_ = 1.5, *p* = 0.14
FTND	0.1±0.3	0.8±1.4	*U* = 132, *p* = 0.1
HADS depression subscale	3.4±2.3	4±2.9	*t*_(35)_ = 0.71, *p* = 0.49
HADS anxiety subscale	6.1±2.7	7.94±2.9	*t*_(35)_ = 2.04, *p* = 0.05
SOGS	0.05±0.2	8.76±2.4	*U* = 0, *p* < 0.001
Pick-up frequency of 0.2€ coin (1–5)	3.2±1.6	3.7±1.5	*U* = 137, *p* = 0.31

*SAI* *sexual arousability inventory* *AUDIT* *alcohol use disorders identification test* *FTND* *fagerstrom test for nicotine dependence* *HADS* *hospital anxiety and depression scale* *SOGS* *south oaks gambling screen*

*Groups were compared using independent sample t-tests for normally distributed variables, and with Mann-Whitney U tests for non-normally distributed variables*.

To assess the subjects’ motivation for money, we asked them about the frequency with which they would pick up a 0.20€ coin from the street on a scale from 1 to 5 (Tobler et al., 2007) and matched the two groups based on this criterion (Table 1). To ensure that all subjects would be in a similar state of motivation to see erotic stimuli, we asked them to avoid any sexual contact during a period of 24 h before the scanning session. Finally, we also sought to enhance the motivation for money by telling subjects that the financial compensation for their participation would add up the winnings accumulated in one of the three runs. For ethical reasons, however, and unbeknownst to the subjects, they all received a fixed amount of cash at the end of the experiment.

All subjects were medication-free and instructed not to use any substance of abuse other than cigarettes on the day of the scan.

### Experimental task

**Figure 1:**
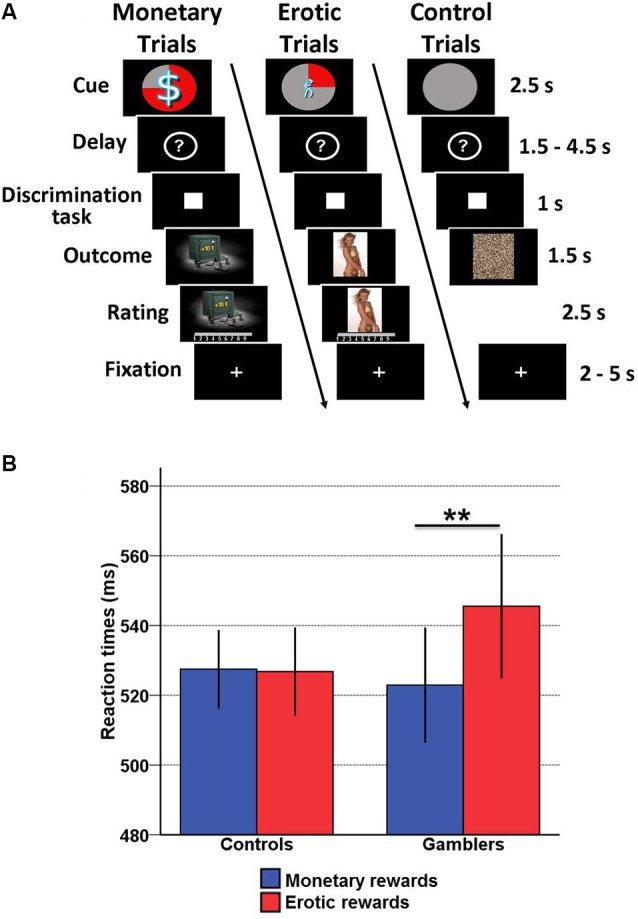
**Incentive delay task and behavioral results. (A)** Subjects first saw a cue informing them about the type (pictogram), intensity (size of pictogram) and probability (pie chart) of an upcoming reward. Three cases are represented here: a 75% chance of receiving a large amount of money (left), a 25% chance of seeing a low erotic content picture (middle) and a sure chance of getting nothing (control trials, right). Then the cue was replaced by a question mark, symbolizing a delay period during which a pseudorandom draw was performed according to the announced probability. Following this anticipation phase, participants had to perform a target discrimination task within <1 s. The target was either a triangle (left button press required) or a square (right button press required). Both their performance and the result of the pseudorandom draw determined the nature of the outcome. In rewarded trials, subjects saw a monetary amount displayed on a safe (high or low amount, left) or an erotic picture (with high or low erotic content, middle), and had to provide a hedonic rating on a continuous scale. In non-rewarded and control trials, subjects saw a scrambled picture (right). **(B)** Plot of mean reaction times according to reward type (monetary/erotic) and group (healthy controls/gamblers) in the discrimination task. There is a significant interaction between group and reward type, driven by slower reaction times for erotic compared to monetary cues in gamblers. Error bars indicate SEM. Asterisks denote significance of Tukey’s HSD tests (** *p* < 0.01).

We used an incentive delay task with both erotic and monetary rewards (Figure 1A). The total number of trials was 171. Each of them consisted of two phases: reward anticipation and reward outcome. During anticipation, subjects saw one of 12 cues announcing the type (monetary/erotic), probability (25/50/75%) and intensity (low/high) of an upcoming reward. An additional control cue was associated with a null reward probability. After a variable delay period (question mark representing a pseudo-random draw), subjects were asked to perform a visual discrimination task. If they answered correctly within less than 1 s, they were then allowed to view the outcome of the pseudo-random draw. In rewarded trials, the outcome was either an erotic image (with high or low erotic content) or the picture of a safe mentioning the amount of money won (high [10/11/12€] or low [1/2/3€]). Following each reward outcome, subjects were asked to provide a hedonic rating on a 1–9 continuous scale (1 = very little pleased; 9 = very highly pleased). In non-rewarded and control trials, subjects were presented with “scrambled” pictures. A fixation cross was finally used as an inter-trial interval of variable length.

### Stimuli

Two categories (high and low intensity) of erotic pictures and monetary gains were used. Nudity being the main criteria driving the reward value of erotic stimuli, we separated them into a “low intensity” group displaying females in underwear or bathing suits and a “high intensity” group displaying naked females in an inviting posture. Each erotic picture was presented only once during the course of the task to avoid habituation. A similar element of surprise was introduced for monetary rewards by randomly varying the amounts at stake (low amounts: 1, 2, or 3€; high amounts: 10, 11, or 12€). The pictures displayed in non-rewarded and control trials were scrambled versions of the pictures used in rewarded trials and hence contained the same information in terms of chromaticity and luminance.

### Plasma cortisol measurements

In order to minimize the effect of circadian hormone rhythms, we conducted all fMRI sessions between 8.50 and 11.45 AM. Just prior to the scanning session, blood samples were collected (mean time, 9.24 AM ± 0.27 mn) to measure the levels of plasma cortisol for each subject. Cortisol concentrations were measured by radioimmunoassay using an antiserum raised in rabbit immunized with cortisol 3-O (carboxy-methyl oxime) bovine serum albumin conjugate, ^125^I cortisol as tracer and buffer containing 8-anilino-1-naphtalene sulfonic acid (ANS) for cortisol-corticosteroid-binding globulin dissociation. Below is the description of the procedure. 100 μL of ^125^I cortisol (10000 dpm) was mixed with the standard or the sample (10 μL), buffer (500 μL) and 100 μL of antiserum solution. Samples were incubated for 45 min at 37°C and 1 h at 4°C. Bound and free cortisol was separated by addiction of a mixture PEG—anti-rabbit gamma globulin. After centrifugation, the radioactivity of the supernatant, containing the cortisol bound to antibody, was counted in a gamma-counter. The within and inter-assay coefficients of variation were less than 3.5 and 5.0% respectively at 300 nmol/L cortisol level. This method has been validated by gas chromatography/mass spectrometry measurements (Chazot et al., 1984).

### Functional magnetic resonance imaging (fMRI) data acquisition

Imaging was conducted on a 1.5 T Siemens Sonata scanner, using an eight-channel head coil. The scanning session was divided into three runs. Each of them included four repetitions of each cue, with the exception of the control condition, repeated nine times. This yielded a total of 171 trials. Within each run, the order of the different conditions was pseudorandomized and optimized to improve signal deconvolution. The order of the runs was counterbalanced between subjects. Before scanning, all subjects were given oral instructions and familiarized with the cognitive task in a short training session. Each of the three functional runs consisted of 296 volumes. Twenty-six interleaved slices parallel to the anterior commissure–posterior commissure line were acquired per volume (field of view, 220 mm; matrix, 64 × 64; voxel size, 3.4 × 3.4 × 4 mm; gap, 0.4 mm), using a gradient-echoechoplanar imaging (EPI) T2*-weighted sequence (repetition time, 2500 ms; echo time, 60 ms; flip angle, 90°). To improve the local field homogeneity and hence minimize susceptibility artifacts in the orbitofrontal area, a manual shimming was performed within a rectangular region including the orbitofrontal cortex (OFC) and the basal ganglia. A high-resolution T1-weighted structural scan was subsequently acquired in each subject.

### Functional magnetic resonance imaging (fMRI) data analysis

The analysis of the data was conducted using Statistical Parametric Mapping (SPM2). The pre-processing procedure included the deletion of the first four functional volumes of each run, slice-timing correction for the remaining volumes and spatial realignment to the first image of each time series. Subsequently, we used tsdiffana utility^1^ to search for residual artifacts in the time series and modeled them with dummy regressors in our general linear model. Then, the functional images were normalized to the Montreal Neurological Institute (MNI) stereotaxic space using the EPI template of SPM2 and spatially smoothed with a 10 mm full-width at half-maximum isotropic Gaussian kernel. Anatomical scans were normalized to the MNI space using the icbm152 template brain and averaged across the subjects. The averaged anatomical image was used as a template to display the functional activations.

Following the preprocessing step, the functional data from each subject was subjected to an event-related statistical analysis. Responses to monetary and erotic cues were modeled separately with 2.5 s box-car functions time-locked to the onset of the cue. For each cue, two orthogonal parametric regressors were added to account for the trial-to-trial variations in reward probability and intensity. The control condition was modeled in a separate regressor. Outcome-related responses were modeled as events time-locked to the appearance of the reward. The two rewards (monetary/erotic) and two possible outcomes (rewarded/non-rewarded) were modeled as four separate conditions. Two covariates linearly modeling the probability and the ratings were further added to each rewarded condition, while another covariate modeling the probability was added to each of the non-rewarded conditions. A last regressor modeled the appearance of a scrambled picture in the control condition. All regressors were subsequently convolved with the canonical hemodynamic response function and entered in a first level analysis. A high-pass filter with a cut-off of 128 s was applied to the time series. Contrast images were calculated based on the parameter estimates output by the general linear model, and were then passed in a second level group analysis.

Second-level analyses focused on the anticipation phase. First, we examined the contrast “monetary > erotic cue” in gamblers minus control subjects. This contrast was thresholded using a cluster-wise family-wise error (FWE) corrected *p* < 0.05. Then, based on our hypothesis, we investigated the relationship between basal cortisol levels and the differential brain response to monetary versus erotic cues. This correlation was computed separately for each group, and was then compared between groups. Based on our *a priori* hypotheses regarding the role of the ventral striatum in attributing incentive salience to reward cues, we used a small volume correction (SVC) based on 7 mm radius spheres centered around the peak voxels reported in a recent meta-analysis on reward processing (*x*, *y*, *z* = 12, 10, −6; *x*, *y*, *z* = −10, 8, −4) (Liu et al., 2011). We used a cluster-wise FWE corrected threshold of *p* ≤ 0.05. To further describe the patterns of activation, we used the EasyROI toolbox to extract the parameter estimates from significant clusters in the ventral striatum.

## Results

### Hormonal data

No significant differences between PGs and healthy control subjects were observed in basal cortisol levels (PGs: mean = 511.59, SD = 137.46; Healthy controls: mean = 588.7, SD = 121.61; *t*_(35)_ = −1.81, *p* > 0.05). This is consistent with findings from recent studies reporting no difference in basal cortisol levels between recreational and PGs (Franco et al., 2010; Paris et al., 2010a,b). In addition, we performed a correlation analysis between cortisol levels and gambling symptom severity in PGs as indexed by the SOGS scale. Our result did not reveal a significant correlation between these variables (*r* = −0.35, *p* = 0.17).

### Behavior

In our previous study (Sescousse et al., 2013), the main behavioral finding was a group × reward type interaction in the reaction time data, reflecting a weaker motivation for erotic compared with monetary rewards in gamblers. Given that one subject was discarded from our current analysis due to a failure to collect hormonal data, we performed this analysis again without this subject. The previous group × reward type interaction remained significant without this subject (*F*_(1, 35)_ = 7.85, *p* < 0.01). In addition, Tukey’s *post-hoc* *t*-tests confirmed that the interaction was due to slower reaction times for erotic (mean = 547.54, SD = 17.22) compared with monetary rewards (mean = 522.91, SD = 14.29) in gamblers relative to healthy controls (*p* < 0.01) (Figure 1B). However, there was no significant correlation between basal cortisol levels and the performance on the discrimination task in either group.

### Brain-cortisol correlation

**Figure 2:**
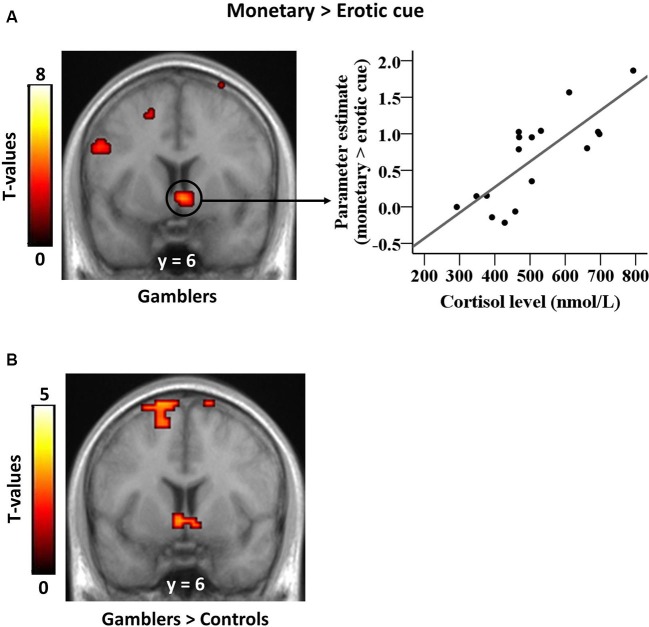
**Correlation between striatal cue reactivity and basal cortisol levels in gamblers. (A)** Ventral striatal responses to monetary versus erotic cues in gamblers are positively correlated with basal cortisol levels. The scatter plot illustrates this positive correlation within significant striatal voxels extracted from the whole-brain map. **(B)** This correlation between ventral striatal responses to monetary versus erotic cues and basal cortisol levels is stronger in gamblers than in healthy controls.

Our previously published analysis revealed a group × reward type interaction in the ventral striatum, reflecting a larger differential response to monetary versus erotic cues in PGs compared with controls (Sescousse et al., 2013). In our current analysis, the results of the group × reward type interaction were still significant after removing the discarded subject (*x*, *y*, *z* = −9, 0, 3, *T* = 4.11; 18, 0, 0, *T* = 3.88; *p*_(SVC)_ < 0.05, FWE). The present analysis focused on how this differential response relates to endogenous cortisol levels. Between-subject correlation analyses revealed a positive relationship between cortisol levels and BOLD responses to monetary versus erotic cues in the ventral striatum of gamblers (*x*, *y*, *z* = 3, 6, −6, *T* = 4.76, *p*_(SVC)_ < 0.05, FWE; Figure 2A), but no such relationship in healthy controls. The direct comparison between groups was also significant (*x*, *y*, *z* = −3, 6, −6, *T* = 3.10, *p*_(SVC)_ ≤ 0.05, FWE; Figure 2B). We additionally examined whether cortisol levels were correlated with brain activity elicited by each reward cue separately, as compared to the control cue. This analysis did not reveal any significant correlation in the ventral striatum in either group (at *p* < 0.001 uncorrected).

## Discussion

To the best of our knowledge, this is the first study exploring the relationship between cortisol levels and brain activation during an incentive delay task in PGs. In line with our *a priori* hypothesis, we observed that higher endogenous cortisol levels were associated with an increased differential neural response to monetary versus erotic cues in the ventral striatum of gamblers as compared to healthy controls. This indicates a specific role of cortisol in biasing gamblers’ motivation towards monetary relative to non-monetary cues. Thus, cortisol may contribute to the addictive process in PGs by enhancing the saliency of gambling-related cues over other stimuli. Because enhanced incentive salience of gambling-related cues in PGs triggers gambling urges, this supports a link between cortisol and PGs’ motivation to pursue monetary rewards.

One potential mechanism through which cortisol might act to influence cue-elicited brain activity is glucocorticoid receptors in the NAcc. It has been shown that glucocorticoid hormones act on the brain through binding with two main intracellular receptors: the mineralocorticoid receptor (MR) and the glucocorticoid receptor. Glucocorticoid hormones play a fundamental role in reward-related behavior *via* their influence on mesolimbic dopamine circuitry and the NAcc in particular. For example, animal evidence shows that glucocorticoid hormones facilitate dopamine transmission in the NAcc shell through glucocorticoid receptors (Marinelli and Piazza, 2002). Microdialysis studies reported that corticosterone has stimulant effects on dopamine transmission in the NAcc (Piazza et al., 1996). Furthermore, infusion of glucocorticoid receptor antagonists has inhibitory effect on drug-induced dopamine release in the NAcc (Marinelli et al., 1998). In line with these findings in animals, human studies found evidence that cortisol levels were positively associated with amphetamine-induced dopamine release in the ventral striatum (Oswald et al., 2005).

It is important to note that we did not observe differences in basal cortisol levels between PGs and controls. Although this finding is in agreement with previous reports showing no difference in basal cortisol levels between PG and recreational gamblers (Meyer et al., 2004; Paris et al., 2010a,b), it does not imply that there is no HPA dysfunction in PGs. Indeed, while most previous studies investigating cortisol levels in PGs have focused on HPA responses to stress-inducing cues, such as gambling cues (Ramirez et al., 1988; Meyer et al., 2000; Franco et al., 2010), in the current study we measured baseline cortisol and its relationship with striatal activations. Moreover, other factors, such as the time of the day when blood or saliva are collected for cortisol level assessment, need to be considered because there are known endogenous diurnal variation in cortisol levels, which may vary between PGs and healthy controls or recreational gamblers. In particular, PGs may have a greater cortisol rise following waking than do recreational gamblers (Wohl et al., 2008).

Another important aspect to consider is that although cortisol is frequently used as a biomarker of psychological stress, a linear relationship between cortisol and other measures of HPA related endocrine signals does not necessarily exist (Hellhammer et al., 2009). Moreover, the absence of relationship between reward-related activity and basal cortisol levels in healthy controls is consistent with the variable effects of both acute stress and cortisol levels observed in the neuroimaging literature on reward processing in healthy individuals. For example, a recent study reported that stress reduces NAcc activation in response to reward cues, but that cortisol suppresses this relationship, as high cortisol was related to stronger NAcc activation in response to reward (Oei et al., 2014). Another study reported that acute stress decreased the response of the dorsal (not ventral) striatum and OFC to monetary outcomes (Porcelli et al., 2012), while no difference was observed in the NAcc between a stress group and control group using an emotion-induction procedure (Ossewaarde et al., 2011). Together, the evidence from fMRI studies indicates non-trivial relationships between stress, cortisol levels and brain activation and suggest that stress and cortisol may play distinct mediating roles in modulating sensitivity to potentially rewarding stimuli through the ventral striatum.

Several limitations of the present study need to be considered. First, only male PG were involved in the current study. It remains unclear whether our current findings would extend to female gamblers. This is an important question because sex differences exist in several aspects of gambling activity (Tschibelu and Elman, 2010; Grant et al., 2012; González-Ortega et al., 2013; van den Bos et al., 2013). Moreover, the modulatory effect of a number of hormonal factors on cognitive functioning varies between sexes (Kivlighan et al., 2005; Reilly, 2012; Vest and Pike, 2013). The current study only included men because they are generally more responsive to visual sexual stimuli than women (Stevens and Hamann, 2012; Wehrum et al., 2013) and show an elevated risk for gambling problems or severity of gambling compared to women (Toneatto and Nguyen, 2007; Wong et al., 2013). Second, we cannot make causal inferences regarding the effects of cortisol on neural responses because our results are based on correlational analyses. A pharmacological design with external cortisol administration compared to a placebo condition would be needed to assess the causal role of cortisol on gambling addiction. Despite these limitations, we believe that our current findings provide a foundation for further research on the interaction between cortisol and brain responses to incentive cues.

## Conclusions

We have found that, in PGs, endogenous cortisol levels are associated with a differential activation of the ventral striatum in response to gambling-related incentives relative to non-gambling-related incentives. Our results point to the importance of integrating endocrinology with a cognitive neuroscience approach to elucidate the neural mechanisms underlying maladaptive gambling behavior. Finally, this study may have important implications for further research investigating the role of cortisol on vulnerability to develop behavioral addictions such as pathological gambling.

## Conflict of interest statement

The authors declare that the research was conducted in the absence of any commercial or financial relationships that could be construed as a potential conflict of interest.

## Footnotes

^1^http://imaging.mrc-cbu.cam.ac.uk/imaging/215DataDiagnostics
